# Elemental bioimaging shows mercury and other toxic metals in normal breast tissue and in breast cancers

**DOI:** 10.1371/journal.pone.0228226

**Published:** 2020-01-31

**Authors:** Roger Pamphlett, Laveniya Satgunaseelan, Stephen Kum Jew, Philip A. Doble, David P. Bishop

**Affiliations:** 1 Discipline of Pathology, Sydney Medical School, Brain and Mind Centre, The University of Sydney, Sydney, New South Wales, Australia; 2 Department of Neuropathology, Royal Prince Alfred Hospital, Sydney, New South Wales, Australia; 3 Department of Tissue Pathology and Diagnostic Oncology, Royal Prince Alfred Hospital, Sydney, New South Wales, Australia; 4 Elemental Bio-Imaging Facility, School of Mathematical and Physical Sciences, University of Technology Sydney, Sydney, New South Wales, Australia; Chinese Academy of Sciences, CHINA

## Abstract

**Objective:**

Exposure to toxic metals such as mercury has been proposed to be a risk factor for the development of breast cancer since some metals can promote genetic mutations and epigenetic changes. We sought to find what toxic metals are present in normal breast tissue and in the tumours of women who had mastectomies for invasive ductal breast carcinoma.

**Materials and methods:**

Formalin-fixed paraffin-embedded blocks from mastectomies for breast carcinoma were examined from 50 women aged 34–69 years. Paraffin blocks selected for elemental analysis were from breast tissue not involved by carcinoma and from the carcinoma itself. Seven micrometer-thick sections were stained with autometallography to demonstrate the presence of mercury, and subjected to laser ablation-inductively coupled plasma-mass spectrometry (LA-ICP-MS) to confirm the presence of mercury and to detect other toxic metals.

**Results:**

Autometallography-detected mercury was seen in intraductal secretions and some luminal epithelial cells of normal breast lobules in 26 (55%) of the 47 samples where lobules were present, and in 10 (23%) of carcinomas from the 44 samples where carcinoma was present. In eight samples ductal carcinoma in situ was present and one of these contained mercury. LA-ICP-MS confirmed the presence of mercury in samples that stained with autometallography, and detected lead, iron, nickel, aluminium, chromium and cadmium in some samples.

**Conclusions:**

Mercury was present in normal breast lobules in more than half of mastectomy samples that contained an invasive carcinoma, and in a smaller proportion of carcinomas and ductal carcinomas in situ. Other toxic metals that may interact synergistically with mercury could be detected in some samples. These findings do not provide direct evidence that toxic metals such as mercury play a role in the pathogenesis of breast cancer, but suggest that future molecular biological investigations on the role of toxic metals in breast cancer are warranted.

## Introduction

Breast cancer is the most common cancer, and the leading cause of cancer death, in women. Many of the risk factors for breast cancer are associated with estrogens, such as early menarche, late menopause, and obesity [1]. Other risk factors include increasing age, hormonal contraception or therapy for the menopause, alcohol, and predisposing germline mutations. However, these factors account for less than half of the overall risk to breast cancer [2,3]. In recent years interest has arisen in the possibility that exposures to environmental pollutants, such as the toxic metals mercury and cadmium, could be risk factors for breast cancer [4–25]. Many of these epidemiological and tissue studies have analysed multiple toxic metals in relation to breast cancer, which is important since synergistic effects between toxic metals in producing cellular damage are increasingly being recognised [26,27].

Toxic metals such as mercury could promote carcinogenesis directly through gene mutations [28–32], epigenetic changes [33], or by acting as estrogen-simulating agents after binding to estrogen receptors [10]. Mercury interferes with DNA repair mechanisms [30,32], and mutations in genes such as *BRCA1* that play a part in DNA repair are associated with breast cancer, suggesting that these environmental toxins and gene mutations may have injury to DNA repair in common [34].

Atmospheric mercury is the toxic metal most strongly associated with breast cancer [24]. Exposure to mercury is common and widespread via the world-wide atmosphere-water-soil mercury cycle [35], and human uptake from fish consumption, dental amalgam tooth fillings, and occupational exposure is well established [36]. Mercury is known to have estrogen-simulating properties [10] as well as the potential to cause genetic mutations [32] and epigenetic changes [37], and mercury can promote the proliferation of breast cancer cells [38]. The amount of mercury in breast milk has been extensively studied because of concerns about transfer of the metal to the fetus [39]. Mercury transporters have been described in human breast glands [40] and these could facilitate the transfer of mercury from the circulation into breast epithelial cells and secretions [41]. Any study of toxic metals in breast cancer therefore needs to include mercury in the range of metals examined.

The findings of epidemiological studies on links between toxic metals and breast cancer have varied and are often contradictory, so other methods to assess toxicant exposures have been attempted, such as measuring metal levels within breast cancer tissue, but these too have yielded diverse results [42–44]. These studies have relied mostly on tissue-destructive techniques such as atomic absorption spectroscopy, so the identity of the cells taking up the metals could not be determined. Furthermore, cancerous tissue could take up toxic metals secondarily to increased cell turnover and blood flow, or due to de novo expression of metal transporters, so the metals detected within carcinomas may not be relevant to the initial cause of the neoplasm. To help overcome these difficulties, we used two techniques, autometallography (AMG) which demonstrates the intracellular presence of mercury, silver and bismuth [45,46], and laser ablation-inductively coupled plasma-mass spectrometry (LA-ICP-MS) which detects multiple elements in tissue sections [47]. We focussed on looking for metals in breast epithelial cells and in the lumens of acini and small terminal ducts (here together termed ‘lobules’) that were unaffected by carcinoma, to get an indication as to whether metals in these sites could initiate neoplasia, since most cancerous lesions appear to arise in these lobules [48–53].

## Materials and methods

### Ethics statement

This study was approved by the Human Research Committee, Sydney Local Health District (Royal Prince Alfred Hospital Zone), in accordance with the Declaration of Helsinki as revised in 2000. The institutional review board waived the need for written informed consent from individuals studied since this was a de-identified retrospective study of archived paraffin-embedded surgical samples. Patient records were searched for age at time of surgery, month and year of surgery (between January 2010 and December 2014), microscopic diagnosis of tumour and non-tumour tissue, tumour grade, presence of ductal carcinoma in situ, and estrogen, progesterone, and human epidermal growth factor receptor status of the carcinoma.

### Tissue samples

Hematoxylin and eosin-stained microscopic slides from the Royal Prince Alfred Hospital tissue repository were examined to find two paraffin tissue blocks that contained (1) normal breast tissue and (2) carcinoma, from 50 women aged between 34–69 years who had mastectomies for invasive ductal breast carcinoma. Paraffin blocks were sectioned at 7 μm with a Feather S35 stainless steel disposable microtome blade and deparaffinised before AMG and LA-ICP-MS analyses. Nottingham tumours grades (I, II and III) were available for all samples. The carcinoma estrogen receptor (ER), progesterone receptor (PR), and human epidermal growth factor receptor 2 (HER2) status had been measured according to the College of American Pathologists Template for Reporting Results of Biomarker Testing of Specimens from Patients with Carcinoma of the Breast.

### Nomenclature

Breast terminal duct-lobular units are focal collections of intralobular terminal ducts and acini within relatively dense fibrous tissue [48]. The small intralobular terminal ducts and their acinar branches were difficult to differentiate from the acini themselves on AMG staining, and so here are grouped together under the term ‘lobules’. The large intralobular terminal ducts and the extralobular terminal ducts are termed ‘terminal ducts’.

### Autometallography (AMG)

Sections were stained for inorganic mercury bound to sulphide or selenide using silver nitrate AMG, which represents the presence of mercury as black grains [45]. AMG is a sensitive photographic technique that can detect as few as 10 mercury sulphide/selenide molecules in a cell [54]. Briefly, sections were placed in physical developer containing 50% gum arabic, citrate buffer, hydroquinone and silver nitrate at 26°C for 80 min in the dark then washed in 5% sodium thiosulphate to remove unbound silver. Sections were counterstained with mercury-free hematoxylin and viewed with bright-field microscopy. Each staining run included a control section of mouse spinal cord where motor neuron cell bodies contained mercury following an intraperitoneal injection of mercuric chloride [55]. Sections were stained with hematoxylin only to act as a control for the AMG. Cells and secretions that stained with AMG were assumed to contain mercury, though not all samples underwent LA-ICP-MS to confirm that mercury was present (see below). The density of lobules in the carcinoma-unaffected breast tissue was estimated qualitatively on a scale of none (0), low (+) or high (++). The proportion of lobules that contained mercury in glands or terminal ducts was estimated as none (0), <5% (+), or ≥5% (++).

### Laser ablation-inductively coupled plasma-mass spectrometry (LA-ICP-MS)

In addition to mercury, AMG can detect inorganic silver and bismuth [45,46]. Therefore, to see if AMG staining in the breast was due to mercury, and to look for other toxic and trace metals, 12 breast sections (9 AMG-positive, 3 AMG-negative) were subjected to LA-ICP-MS for mercury (Hg), silver (Ag), bismuth (Bi), phosphorus (P, to assess cell density), aluminium (Al), gold (Au), cadmium (Cd), chromium (Cr), iron (Fe), nickel (Ni) and lead (Pb). Analyses were carried out on an NWR193 excimer laser (Kenelec Scientific) hyphenated to an Agilent Technologies 7700 ICP-MS fitted with ‘s’ lenses for enhanced sensitivity, with argon used as the carrier gas. LA-ICP-MS conditions were optimised on NIST 612 Trace Element in Glass CRM and the sample was ablated with a 50 μm spot size and a scan speed of 100 μm/s at a frequency of 20 Hz. The data were collated into a single image file using in-house developed software and visualised using FIJI. Limits of detection for mercury using LA-ICP-MS have been estimated to be between 0.05 and 0.81 μg/g [56].

## Results

### Autometallography

#### AMG in lobules and terminal ducts

**Fig 1:**
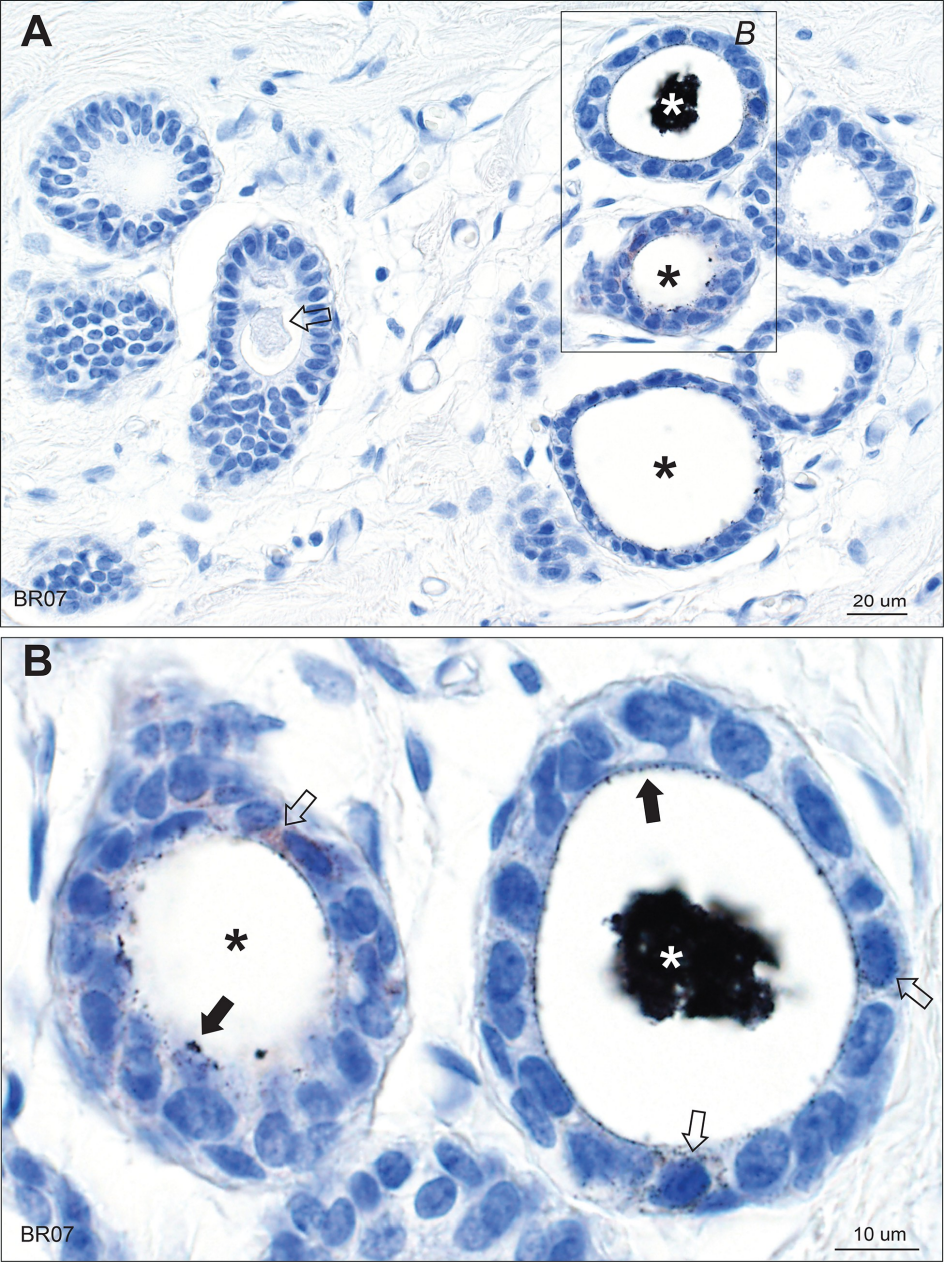
Mercury in breast lobules. (A) One lobule in the group on the right shows black mercury-stained secretion (white asterisk), and two (black asterisks) show mercury on the luminal surface of epithelial cells. The lobules on the left show no mercury, with unstained luminal secretion visible in one (arrow). (B) Enlarged image of the box in A. The lobule on the right has mercury in its artefactually-shrunken luminal secretion (white asterisk). Some mercury-stained secretion is still attached to the luminal surface of the epithelial cells (solid arrows). Fine particulate mercury staining is present in some epithelial cells (open arrows). In the lobule on the left (black asterisk) much of the secretion has fallen out during processing, but mercury-stained secretion remains attached to the luminal surface of some epithelial cells (closed arrow) and is also present in some epithelial cells (open arrow). AMG/hematoxylin. BR: sample identity number.

**Fig 2:**
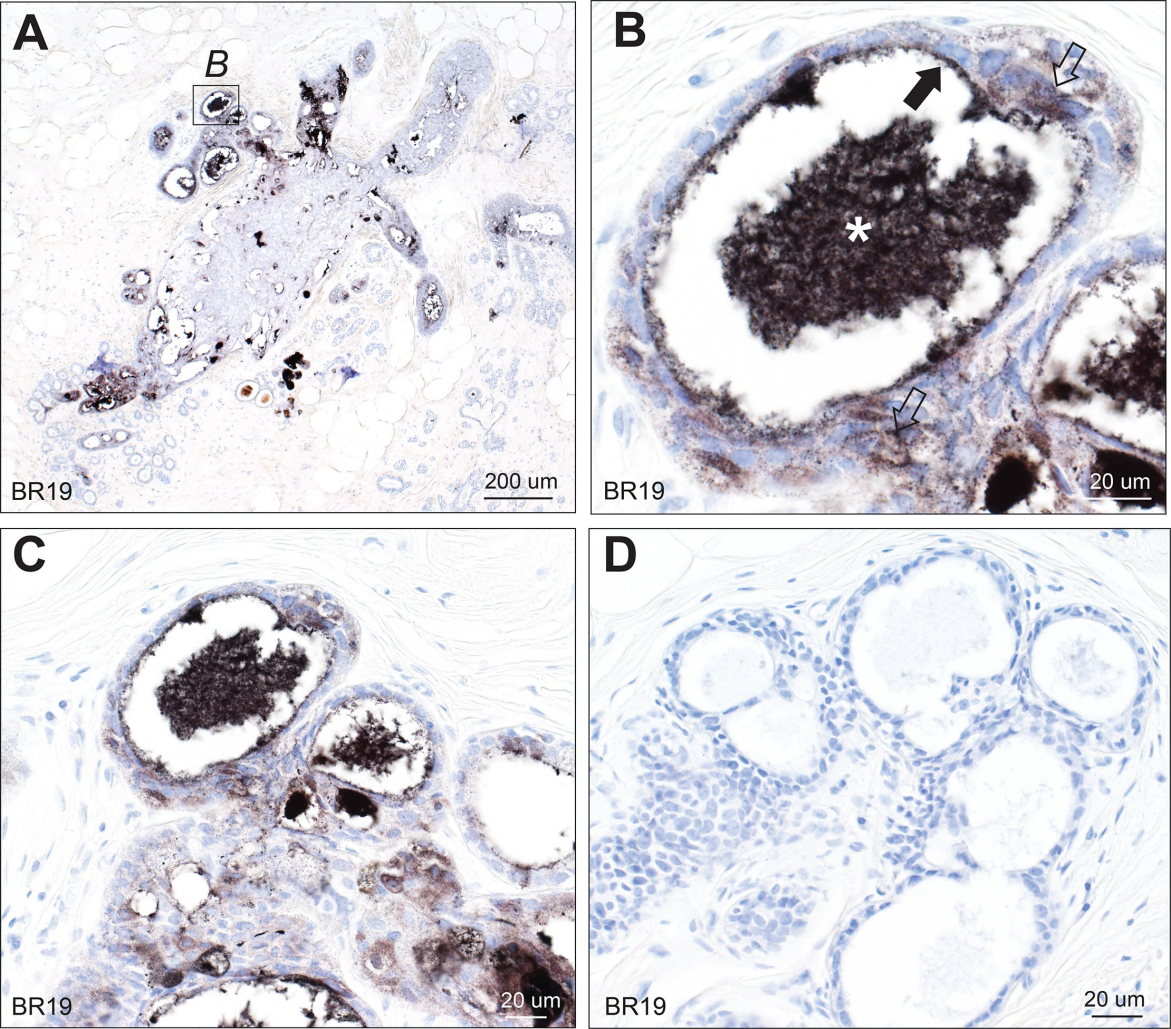
Mercury in breast lobules. (A) Mercury is present in the lumen of many, but not all, lobules. AMG/hematoxylin. (B) Magnified view of the box in A shows mercury within the shrunken luminal secretion (asterisk) of this lobule, with some remaining mercury on the luminal surface of the epithelial cells (solid arrow), and finely dispersed mercury within epithelial cells (open arrows). AMG/hematoxylin. (C, D) The black mercury staining in multiple lumens and cells of these lobules (C) in this AMG/hematoxylin section can be compared to the absence of black staining in a nearby section stained with hematoxylin only (D). BR: sample identity number.

**Fig 3:**
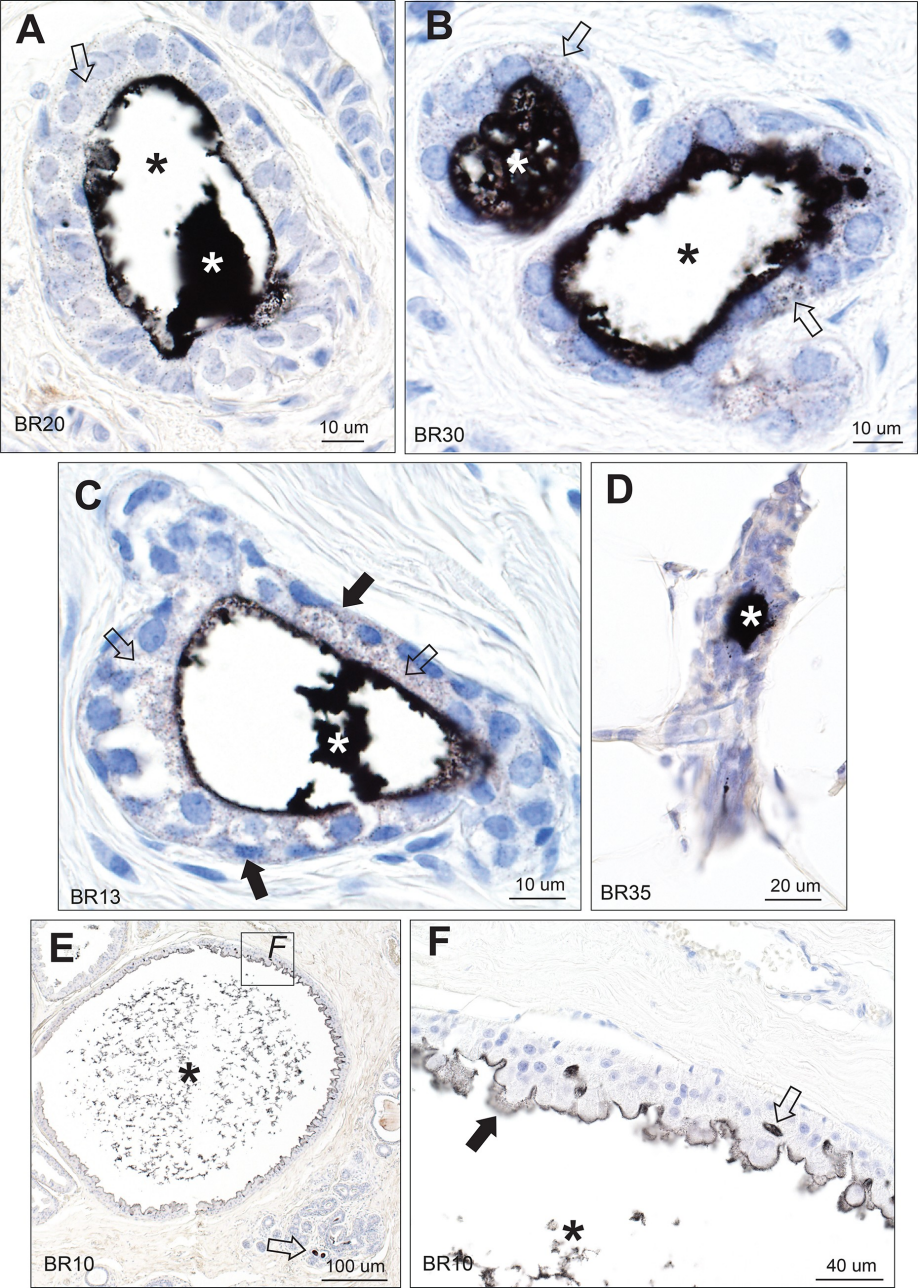
Mercury in breast lobules. (A, B, C) Mercury is present in the luminal secretion (white asterisks) and epithelial cells (open arrows) of these lobules from three different tissue samples. In A and B, much of the luminal secretion has artefactually dropped out (black asterisks). In C, some mercury may also be present in the cytoplasm of myoepithelial cells (solid arrows) as well as in epithelial cells (open arrows). (D) Mercury is present in the luminal secretion (asterisk) of this atrophic lobule, with surrounding fatty tissue. (E) A large lobule with apocrine metaplasia is filled with fragmented secretion that stains for mercury (asterisk). Some adjacent normal-sized lobules contain mercury in their lumens (arrow). (F) Enlarged view of the box in E shows mercury in the remaining luminal secretion (asterisk), on the luminal surface of apocrine cells (solid arrow), and between apocrine cells (open arrow). AMG/hematoxylin. BR: sample identity number.

**Fig 4:**
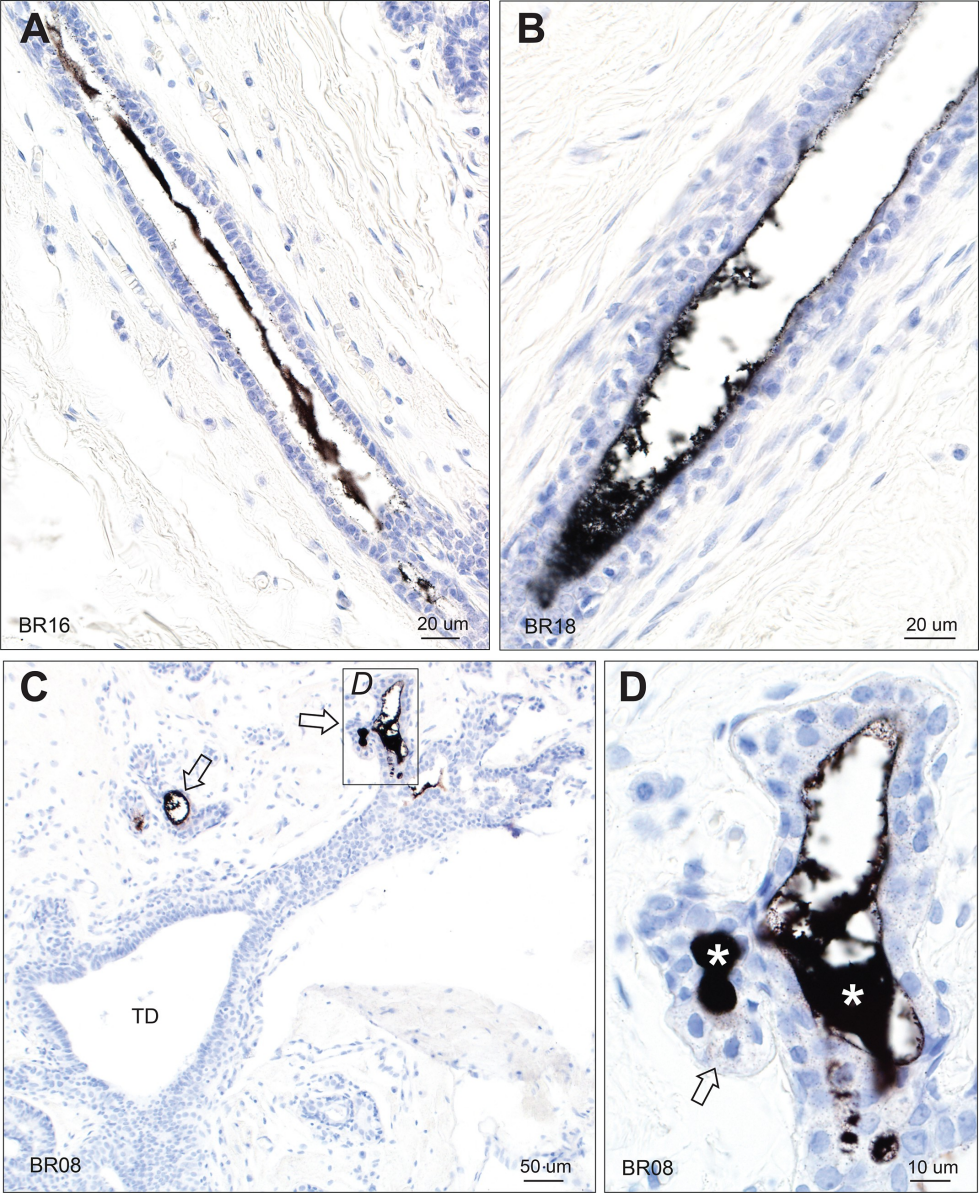
Mercury in breast terminal ducts and lobules. (A, B) Mercury is present within the luminal secretion of these extralobular terminal ducts, but not within the luminal epithelial cells. (C) No mercury is seen in the intralobular terminal duct (TD) of this breast, despite mercury being present in adjacent lobules (arrows). (D) Enlarged view of the box in C shows mercury in the luminal secretion (asterisks) and as fine black particles in epithelial cells (eg, arrow) of this lobule. AMG/hematoxylin. BR: sample identity number.

Mercury in breast secretions was identified by black AMG staining within the lumens of lobules and terminal ducts (Figs 1–4). Control hematoxylin-only staining of sections that contained mercury-positive lobules on AMG showed no black grains (Fig 2). Often secretions had fallen out of lumens during processing, or shrunken away from luminal epithelial cells, leaving a fragmented peripheral rim of secretion (Figs 1–3). Non-AMG-stained luminal secretions were often present in lobules immediately adjacent to mercury-containing lobules (Fig 1). Even in atrophic glands within fatty tissue, mercury could be seen in some luminal secretions (Fig 3). Some dilated lobules that had undergone apocrine metaplasia contained mercury in their lumens, and mercury could be seen between apocrine cells (Fig 3). In other samples, lobules with apocrine metaplasia were mercury-free, even when nearby lobules contained mercury.

About one-fifth of lumens with mercury-containing secretions had fine granular mercury staining within the cytoplasm of luminal epithelial cells. This mercury often surrounded the epithelial cell nucleus (Figs 1–4). Faint mercury staining was seen in the cytoplasm of some myoepithelial cells (Fig 3), though these were often difficult to separate from luminal epithelial cells. Mercury was not seen in luminal epithelial cells in the absence of mercury in adjacent secretions. Of the 26 samples that contained mercury-positive lobules, the proportion that had any mercury in lobules was <5% in 17 samples (65%), and ≥5% in 9 samples (35%), indicating that most samples contained only a small proportion of lobules with mercury. Mercury was seen in the luminal secretions of a few extralobular terminal ducts, but not within their lining epithelial cells (Fig 4). No mercury was seen in the lumen or epithelial cells of large intralobular terminal ducts (Fig 4).

#### Mercury in lobules in relation to tumour characteristics

The ages of the patients, the density of lobules, the proportion of lobules, carcinomas and ductal carcinomas in situ (DCIS) with AMG staining, and the carcinoma biomarkers are shown in S1 Table. In three normal breast tissue samples the paraffin section contained only fat and no lobules. In six carcinoma samples the tumour size was too small to reliably evaluate the metal content.

The relation of mercury in normal breast lobules to age and tumour characteristics are shown in Table 1. Contingency 2x2 analyses with Fisher’s exact tests were undertaken, with statistical significance at the 0.05 level. Women with mercury in their breast lobules tended to be older (65%) than younger (46%), have a higher (65%) than a lower (46%) tumour grade, and to have a PR-negative (77%) rather than a PR-positive (47%) tumour, though none of these differences was statistically significant. No relationship existed between breast lobule mercury and lobule density or tumour ER status.

**Table 1:** Presence of mercury in lobules compared to age and tumour characteristics.

	Mercury in lobules		
Characteristic	Positive No. (%)	Negative No. (%)	p
Total	26 (55)	21 (45)	
Age 34–55 years	11 (46)	13 (54)	0.24
Age 56–69 years	15 (65)	8 (35)	
Lobule density low	13 (50)	13 (50)	0.56
Lobule density high	13 (62)	8 (38)	
Tumour grade I & II	11 (46)	13 (54)	0.24
Tumour grade III	15 (65)	8 (35)	
ER Positive	22 (55)	18 (45)	>0.99
ER Negative	4 (57)	3 (43)	
PR Positive	16 (47)	18 (53)	0.1
PR Negative	10 (77)	3 (23)	

#### AMG in neoplastic cells

**Fig 5:**
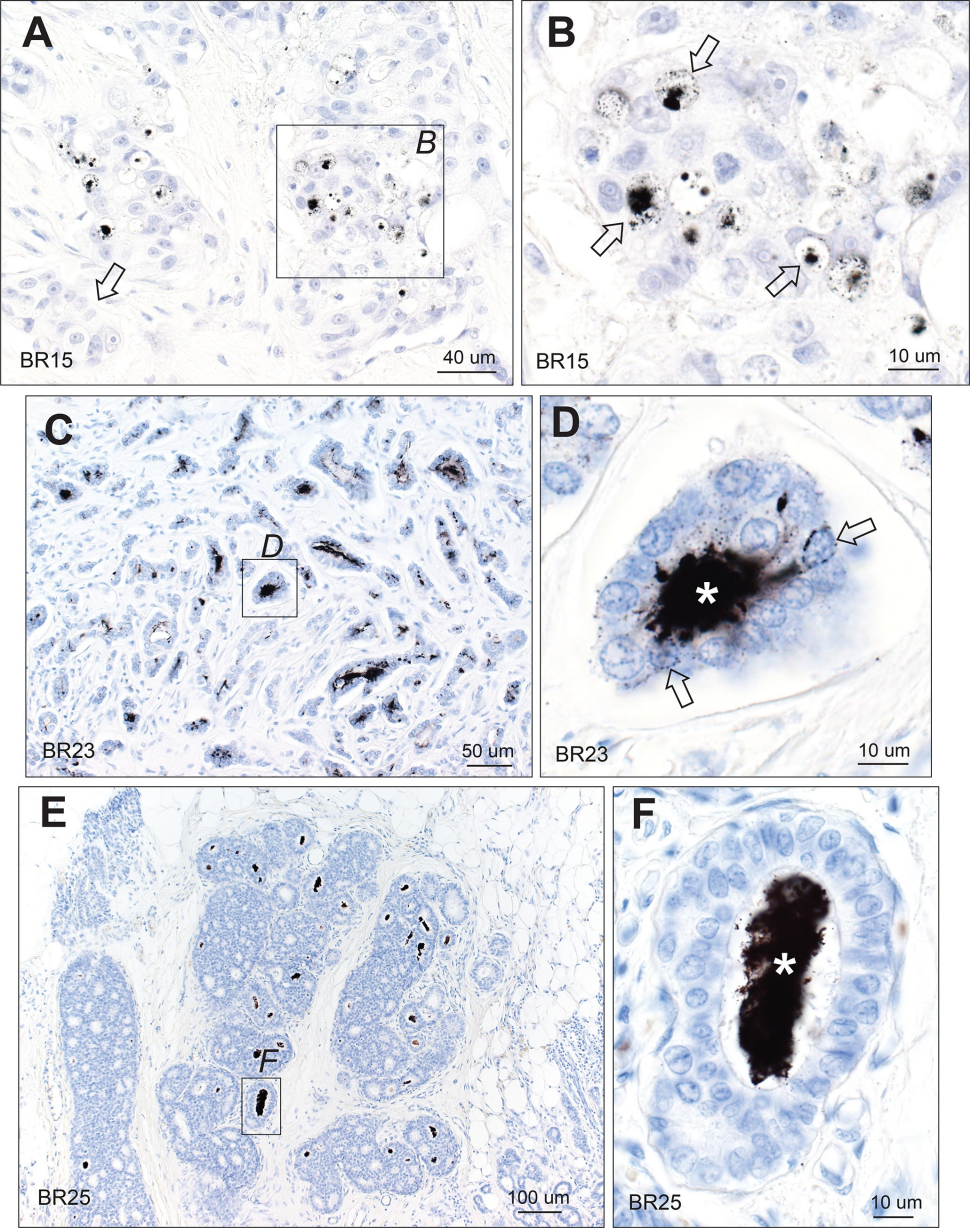
Mercury in breast carcinomas and ductal carcinoma in situ. (A) Mercury is seen within the lumens of carcinoma ducts (eg, within the box), whereas other regions of the carcinoma are mercury-free (eg, arrow). (B) Enlarged view of the box in A shows mercury in carcinoma ducts (eg, arrows). (C) Numerous ducts of this carcinoma contain mercury within their lumens (eg, in box). (D) Enlarged view of the box in C shows mercury in the carcinoma-cell-lined lumen of this duct (asterisk), and in adjacent carcinoma cells, where the mercury is often attached to the cell nuclear membrane (eg, arrows). (E) Ductal carcinoma in situ shows many ducts containing mercury (eg, in box). (F) Enlarged view of the box in E shows mercury within a carcinoma-cell-lined lumen (asterisk), but not within adjacent carcinoma cells. AMG/hematoxylin. BR: sample identity number.

Mercury was seen in carcinomas in 10 (23%) of the 44 samples where carcinoma was present. The mercury was usually within the lumen of carcinoma ducts. Mercury could be found within the carcinoma cells surrounding some of these lumens (Fig 5).

#### Ductal carcinoma-in-situ (DCIS)

In eight samples, foci of DCIS were present (S1 Table). In seven of these, no mercury was seen, despite two having nearby carcinomas with mercury deposits. In one sample, both the DCIS (Fig 5) and the adjacent carcinoma contained mercury.

#### Laser ablation-inductively coupled plasma-mass spectrometry (LA-ICP-MS)

LA-ICP-MS was carried out on seven samples containing normal breast tissue (four lobule AMG-positive, three lobule AMG-negative) and on five carcinomas (all AMG-positive).

#### Confirmation of AMG-detected mercury

**Fig 6:**
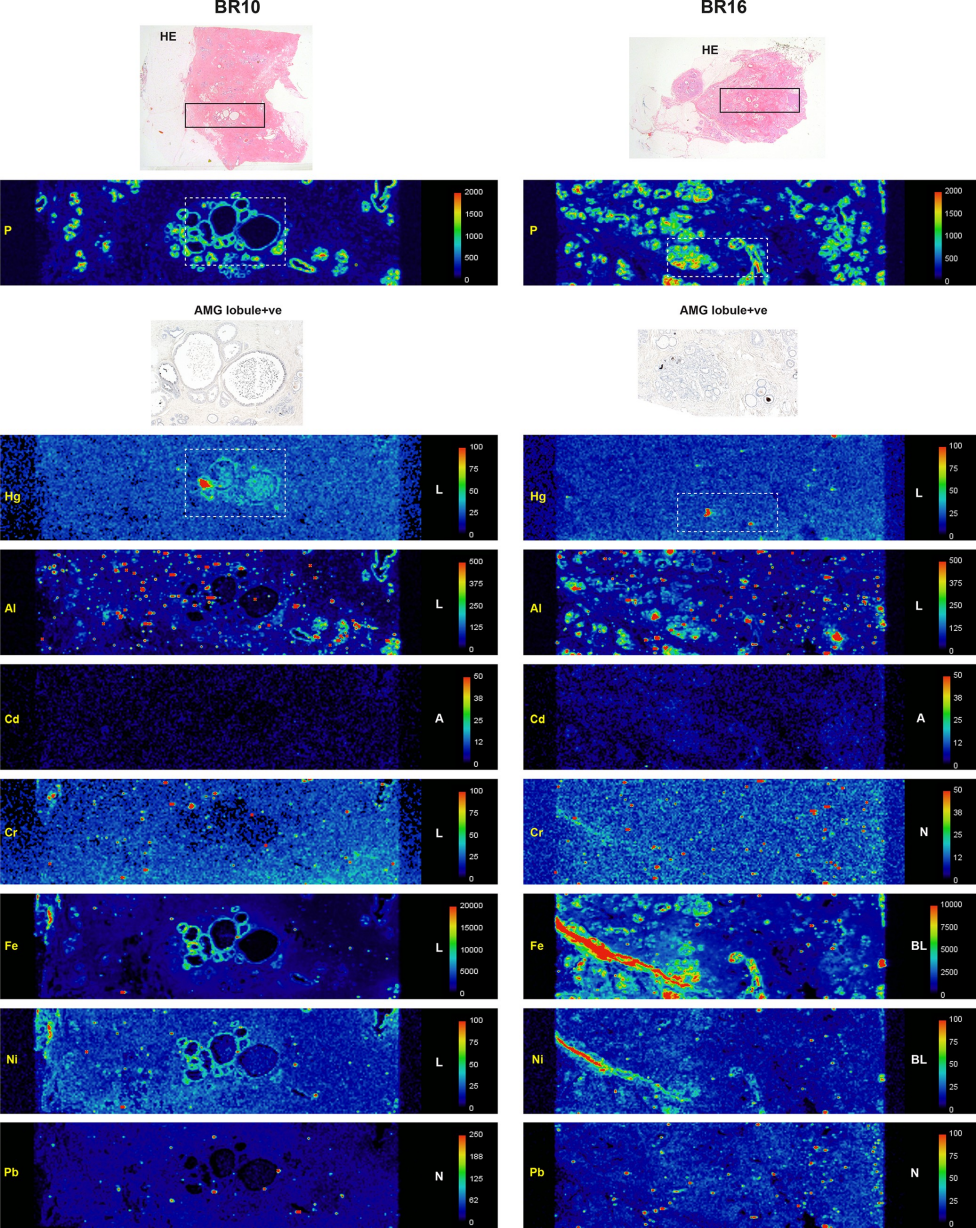
LA-ICP-MS of two AMG-positive breast lobules. Boxes in the hematoxylin-eosin (HE) stained sections in the upper row show the region of LA-ICP-MS analysis. Phosphorus (P) abundance indicates cellular density. Selected regions stained with autometallography (AMG) are indicated in the LA-ICP-MS mercury (Hg) images in dashed boxes. Element distribution = A: absent, B: blood; L: lobules, N: non-localising, S: stroma. Scale = counts per second (proportional to abundance).

**Fig 7:**
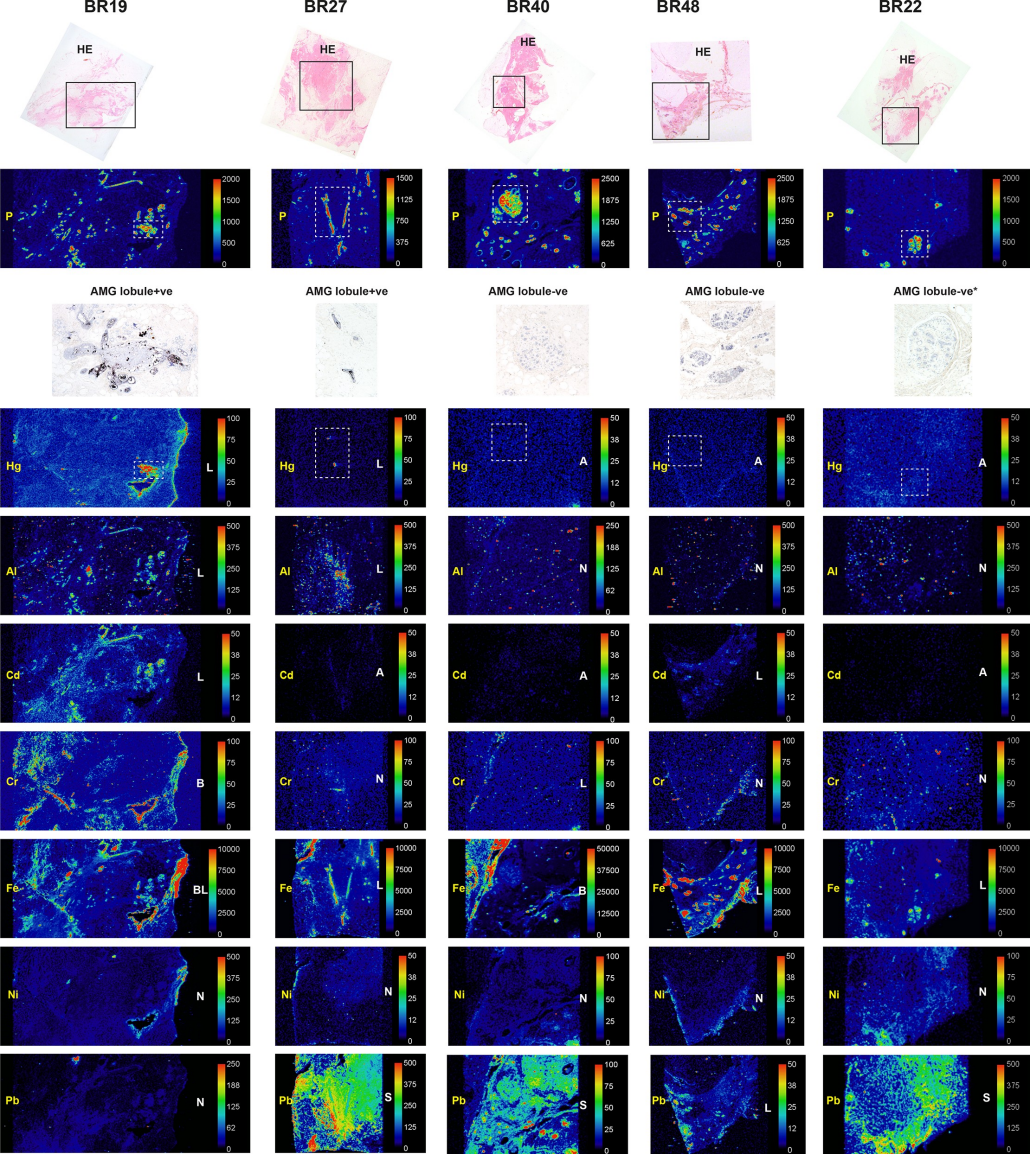
LA-ICP-MS of two AMG-positive and three AMG-negative breast lobules. Boxes in the hematoxylin-eosin (HE) stained sections in the upper row show the region of LA-ICP-MS analysis. Phosphorus (P) abundance indicates cellular density. Selected regions stained with autometallography (AMG) are indicated in the LA-ICP-MS mercury (Hg) images in dashed boxes. Element distribution = A: absent, B: blood; L: lobules, N: non-localising, S: stroma. Scale = counts per second (proportional to abundance). *Sampled in an AMG-negative region.

**Fig 8:**
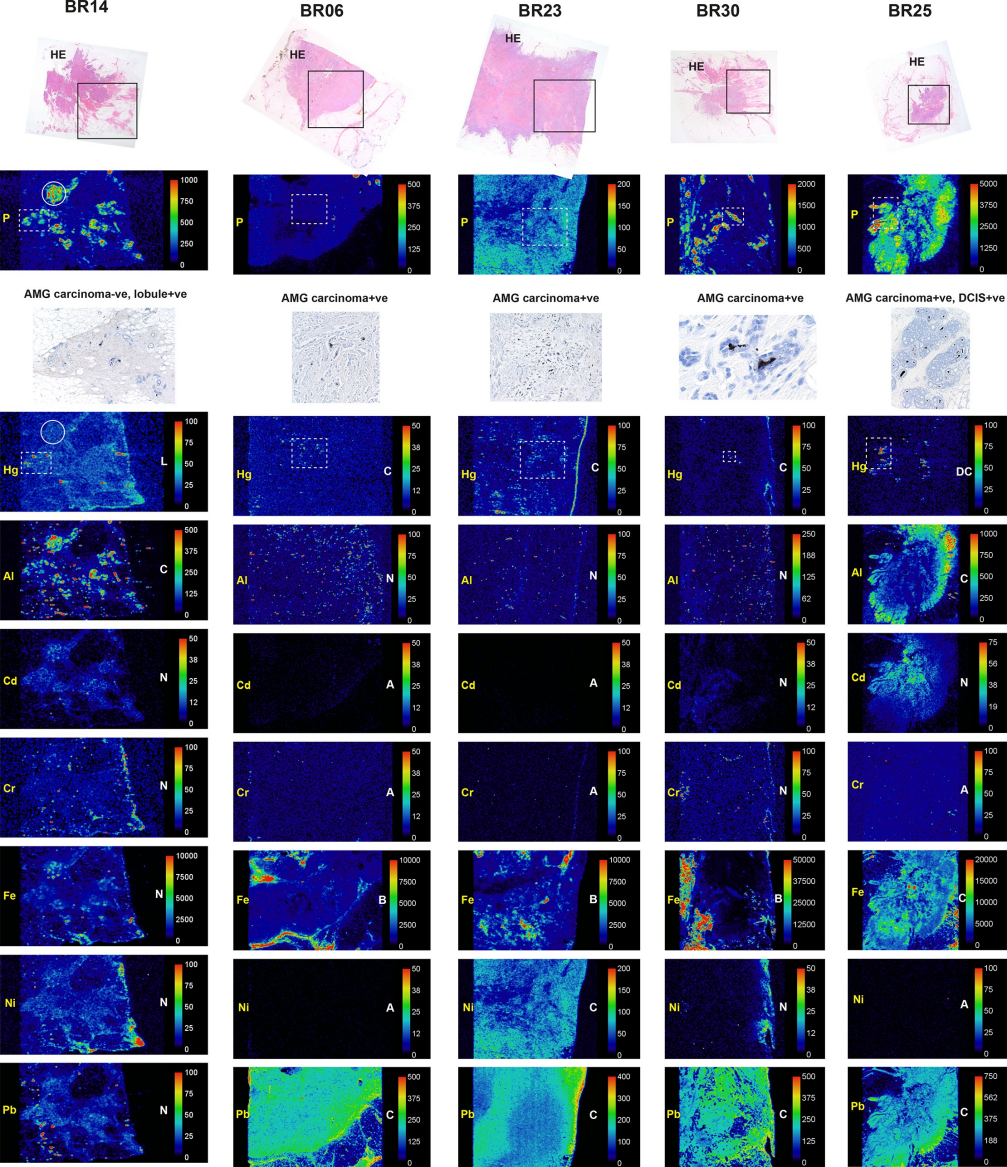
LA-ICP-MS of one AMG-negative and four AMG-positive breast carcinomas. Boxes in the hematoxylin-eosin (HE) stained sections in the upper row show the region of LA-ICP-MS analysis. Phosphorus (P) abundance indicates cellular density. Selected regions stained with autometallography (AMG) are indicated in the LA-ICP-MS mercury (Hg) images in dashed boxes. In the AMG-negative BR14 carcinoma, adjacent lobules (eg, in the dashed box) contain mercury; the carcinoma (eg, in the circle) contains aluminium but no mercury. In BR25 both the DCIS (in the dashed box) and the carcinoma contain mercury; the carcinoma also contains aluminium, iron and lead. Element distribution = A: absent, B: blood; C: carcinoma, D: DCIS, L: lobules, N: non-localising. Scale = counts per second (proportional to abundance).

Mercury was detected in all ten AMG-positive lobule, carcinoma, and DCIS samples, but not in the three AMG-negative lobule samples or in the AMG-negative carcinoma sample (Figs 6–8). Mercury-positivity on LA-ICP-MS correlated best with AMG staining when AMG was present in several closely adjacent lobules or carcinoma ducts. Isolated foci of AMG-positivity (for example, in one small lobule) were not always detectable as mercury on LA-ICP-MS.

#### Non-mercury metals

Non-mercury metals were detected on LA-ICP-MS in some normal breast lobules, some normal stroma and some carcinomas (Figs 6–8). Metals could be localised to normal breast lobules or stroma, to carcinomas, or to blood vessel lumens containing red blood cells. *Cadmium* was seen in a few lobules. *Chromium* was seen occasionally in lobules and in blood vessels. *Iron* was seen often in lobules and blood vessels, and in carcinomas. *Lead* was found occasionally in stroma or lobules, and often in carcinomas. *Nickel* was present in a few lobules, blood vessels and in carcinomas. *Aluminium* was localised in some lobules, and in some carcinomas.

A non-specific background scattering or edge effect of *aluminium*, *bismuth*, *cadmium*, *chromium*, *gold*, *lead*, *nickel* and *silver* was seen in some samples, possibly due to contamination from elements introduced during formalin fixation and processing of tissue for paraffin embedding; of these elements, only chromium is present in the stainless steel blades used for microtomy, so these elements were not introduced at the section cutting stage. To eliminate from consideration potentially-contaminating elements, only elements localised to breast lobules, tumour cells, stroma or blood vessels were considered to be biologically significant. *Bismuth*, *gold* and *silver* were not localised to any of the breast compartments (S1 Fig).

## Discussion

Key findings of this study were that mercury was present in scattered normal breast lobules from about half of individuals who had a mastectomy for breast cancer. Mercury was found in about a quarter of the breast carcinomas from these individuals. Several other toxic metals were found in breast lobules and carcinomas, some of which may act synergistically with mercury to increase its genotoxic effects [27]. Although these findings do not provide direct evidence that toxic metals such as mercury play a role in the pathogenesis of breast cancer, they suggest that future molecular biological investigations on the role of toxic metals in breast cancer are warranted.

**Fig 9:**
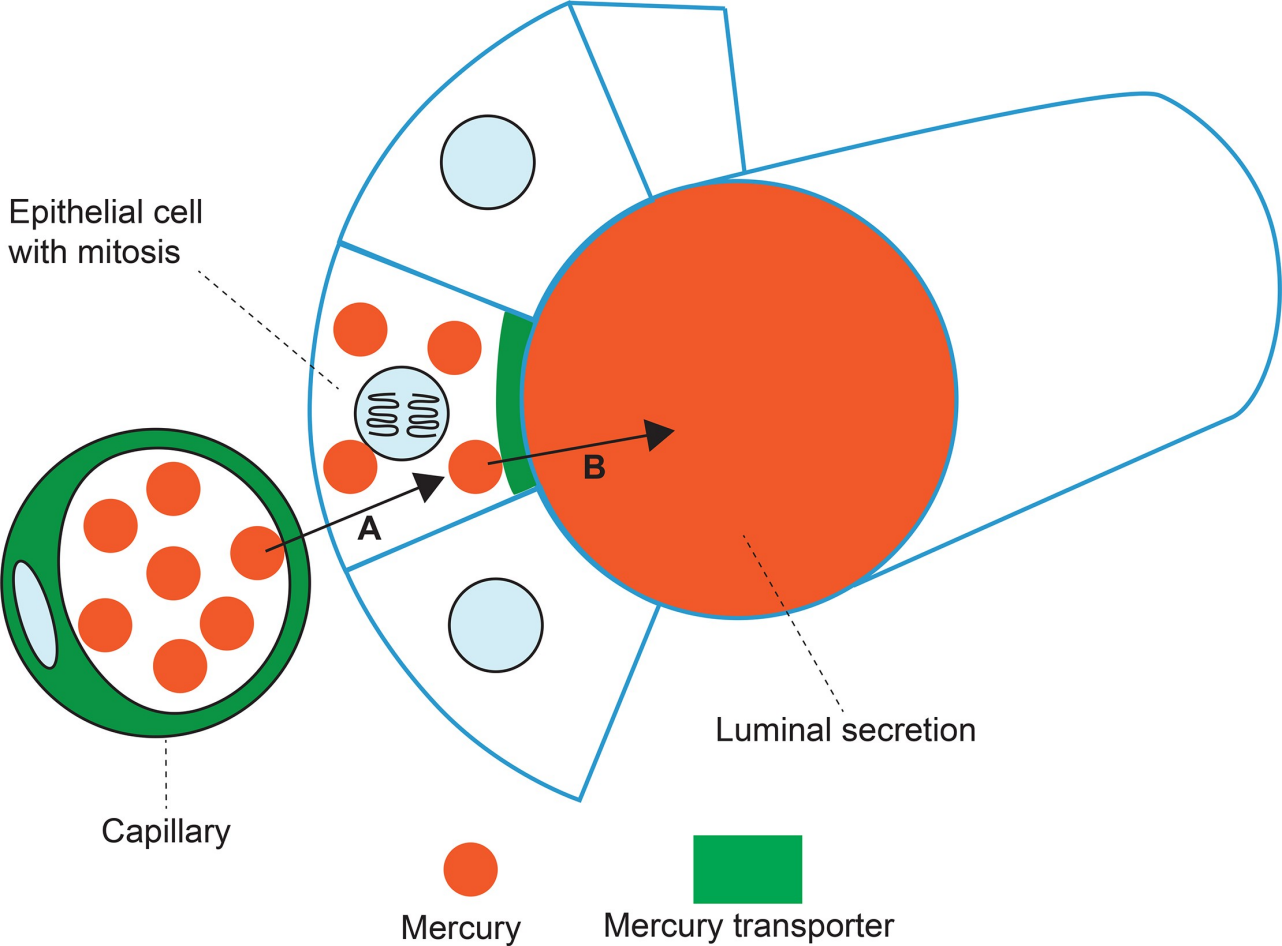
Proposed transfer of circulating mercury through breast epithelial cells into ductules. (A) A capillary with mercury transporters transfers circulating mercury into a luminal epithelial cell. (B) An epithelial cell with apical mercury transporters transfers mercury into the lumen of the breast ductule. Luminal epithelial progenitor cells that are undergoing mitoses may be particularly vulnerable to the genotoxic effects of mercury.

Mercury was usually present in only a small percentage of lobules within individual samples. This is probably because mercury transporters (such as breast cancer resistance protein, BRCP/ABCG2) are normally found in many capillaries, but in only a few lobules, in the human breast [40]. It is likely that mercury transporters are required to transfer circulating mercury through the breast capillaries into the luminal epithelial cells, from which the mercury is then transported into the lumen (Fig 9). It has been proposed that the export protein BRCP aids the secretion of nutrients into the milk, but that the risk of contaminating the milk with xenotoxins is usually low [41]. Although much is known about the mechanism of mercury transporters such as BRCP in renal tubules [57], little detail is currently known about these transporters in the human breast, for example, as to how mercury crosses the basal membrane of ductule epithelial cells.

**Fig 10:**
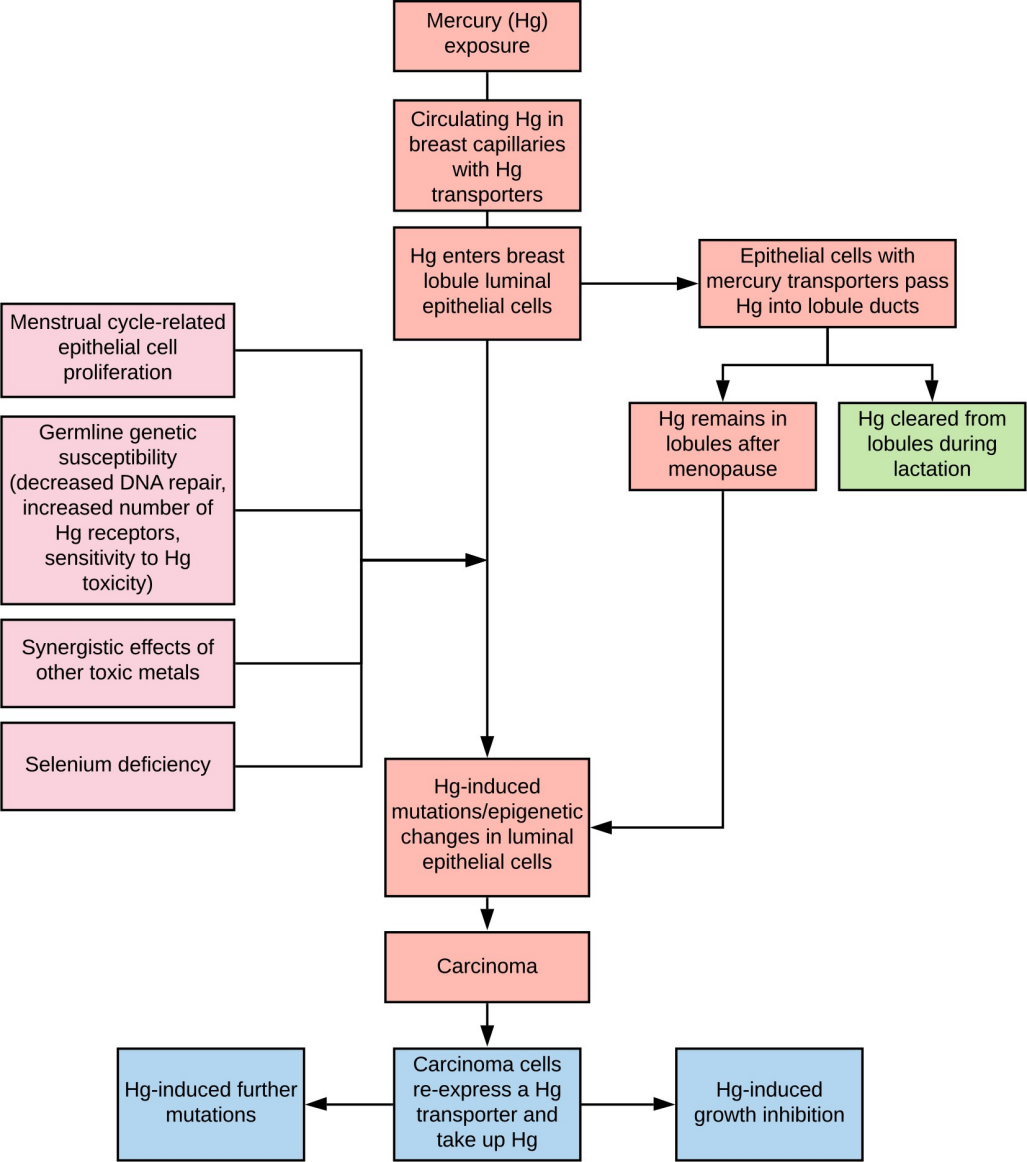
Potential pathogenetic role of mercury in breast cancer. Circulating mercury enters breast epithelial cells and then into the ducts. Predisposing factors for mercury being able to induce cancer-inducing genetic and epigenetic changes include the presence of mitoses in mercury-containing epithelial cells, germline genetic susceptibilities to impaired DNA repair or mercury toxicity, additional toxic metals, and a lack of selenium. Mercury is likely to remain in lobules after menopause, with ongoing susceptibility of breast epithelial cells to neoplastic change. Lactation could decrease the risk of mercury-induced breast cancer by clearing the lobules of mercury-containing luminal secretions. Mercury within carcinoma cells could initiate further mutations, or inhibit cell proliferation by direct cellular toxicity.

The luminal epithelial progenitor cell is the proposed cell of origin for most breast cancers [48–53]. During the menstrual cycle, proliferating luminal epithelial cells with their frequent mitoses are likely to be particularly susceptible to the genotoxic effects of mercury [30,32] (Figs 9 and 10). Therefore, the greater the number of menstrual cycles, the more opportunities mercury would have to damage the DNA of these epithelial cells. This could be one factor contributing to the association between the numbers of menstrual cycles and the risk of breast cancer [58].

Mercury persisted in luminal secretions in some of our post-menopausal breast samples, and the proximity of these secretions to the luminal epithelial cells would ensure continual exposure of these cells to mercury (Fig 10). On the other hand, during lactation much of the mercury in the luminal secretions would be removed, which could explain the decreased risk of breast cancer following lactation [1] (Fig 10).

Human exposure to environmental sources of mercury, for example from seafood consumption or dental amalgam fillings, is common [59]. Furthermore, mercury can frequently be found in the tissues of a general adult human population [47,60]. It is therefore likely that several susceptibility factors are needed for mercury-induced breast carcinoma to occur (Fig 10). These could include germline mutations in genes controlling DNA repair [1], since mercury can damage DNA directly [30,32]. The inheritance of polymorphisms influencing the number of mercury transporters in the breast [61], or of polymorphisms affecting sensitivity to mercury toxicity [62], could also increase the risk of mercury-induced breast cancer. Furthermore, some of the non-mercury metals we found in the breast could act synergistically with mercury to increase its toxicity [26]. Low tissue selenium levels could further increase the intracellular toxicity of mercury [63].

Mercury within carcinomas was usually found in only a small proportion of tumour cells, probably in relation to mercury transporters such a breast cancer resistance protein being expressed in these cells [64]. Mercury could affect the proliferation of carcinoma cells [38] by increasing numbers of abnormal mitoses, or could decrease tumour growth by direct toxicity to tumour cells via mechanisms such as the production of reactive oxygen species [8].

Non-mercury metals in our breast samples that have previously been implicated in the pathogenesis of breast cancer were aluminium [43,65–67], cadmium [10,24,68], iron [69,70], nickel [68], chromium [10] and lead [24,71]. However, we were unable to confirm the cellular location of these metals in our samples since reliable histochemical methods are not available to detect them, and cell-specific elemental techniques such as x-ray fluorescence microanalysis require the use of frozen sections. Some of these metals could interact with mercury to increase its toxicity since synergistic actions between various metals are now recognised [27]. Another way multiple toxic metals could act together to cause breast cancer is if the metals located to different tissue types within the breast. For example, in some of our samples mercury was present in lobule epithelial cells, and lead was present in the stroma, both cell lineages that have been implicated in the pathogenesis of breast cancer [58].

Previous workers comparing exposure to metallic air pollutants to breast tumour estrogen and progesterone receptor status have reported either that arsenic and cadmium exposure increase ER/PR-negative cancer risk [12], that mercury, antimony and cobalt increase ER-positive cancer risk [24], and that cadmium, antimony and cobalt increase ER/PR-negative cancer risk [22]. These studies contained large numbers of subjects, so that the typically small proportion of breast cancers that have combined ER/PR-negative tumours could be assessed statistically. In our smaller sample of 50 tumours only one was ER/PR-negative, so we analysed breast lobule mercury in relation to ER and PR receptor status separately. Mercury in breast lobules did not increase ER-positive cancer risk, but mercury in the breast tended to increase PR-negative cancer risk. This implies that breast mercury may be a more important predictor of PR than ER receptor status, though larger numbers of breast tumour samples would be needed to confirm this.

This study has several limitations. (1) Autometallography demonstrates inorganic mercury bound to sulphides and selenides, but not organic mercury. However, the Hg^++^ cation from the mercurous, mercuric, and vapor forms of mercury is the proximate toxic form of the metal in tissues, and methylmercury is slowly converted to inorganic mercury in the body [59], so inorganic mercury is the most relevant type to detect. (2) We did not have access to breast tissue from individuals without breast cancer, since cosmetic breast reductions are uncommon in public hospitals, and do not usually cover our wide age range. It is also rare to remove breast tissue at routine autopsies. We suspect, however, that similarly high proportions of people without breast cancer would have mercury-containing lobules. This is because mercury is found often in the tissues of women over the ages of 20 years [47,60] and circulating mercury is concentrated in breast milk [39,72]. The finding that mercury is commonly present in human tissues reinforces the concept that other susceptibilities would be needed for mercury to initiate breast cancer. One way to get around the difficulty of findings suitable control breast tissue would be to look at mercury levels in nipple aspirates from women with and without breast cancer [73]; however, in our study usually only a few lobules contained mercury, so this technique might not be sensitive enough to find differences between groups. Ideally, a non-invasive method of imaging mercury in ambulant individuals both with and without breast cancer would be undertaken, but such a technique is not yet available. (3) We did not have individual histories of occupations, seafood consumption or numbers of mercury amalgam dental fillings, so we were unable to estimate exposure to these sources of mercury. A study using questionnaire data on previous mercury exposure [74] to compare people with and without breast cancer would be of interest. (4) Luminal secretions often fell out of the sections during processing. We could infer the presence of luminal mercury on autometallography from fragments of secretion attached to luminal epithelial cells, but this dropout of secretions suggests we probably underestimated the amount of mercury in our LA-ICP-MS samples.

In conclusion, a large proportion of breast samples taken at mastectomy for carcinoma had normal breast lobules that contained mercury, as well as other toxic metals. A smaller proportion of breast carcinomas contained mercury and other toxic metals. Although our results do not provide an unequivocal link between mercury in normal breast lobules and breast cancer, it would seem prudent to limit exposure to mercury as much as possible by restricting the intake of larger predatory fish and considering alternatives to mercury-containing amalgam fillings [59], and to ensure an adequate intake of dietary selenium to help counter the toxic effects of mercury [63].

## Supporting information

S1 Table Characteristics of 50 breast tissue samples. (DOCX)

S1 Fig LA-ICP-MS of silver, gold and bismuth in 12 samples without breast tissue localisation. Breast samples showing the absence of localised staining for Ag, Au and Bi. Phosphorus (P) staining indicates cellular density. Selected regions stained with autometallography (AMG) are indicated in the dashed boxes. Element distribution = A: absent, N: non-localising. Scale = counts per second (proportional to abundance).
(TIF)
