# Supplementary material for: Elemental bioimaging shows mercury and other toxic metals in normal breast tissue and in breast cancers
Source: PLoS One. 2020 Jan 31;15(1):e0228226. doi: 10.1371/journal.pone.0228226 (PMC6993973; doi:10.1371/journal.pone.0228226)
Supplement: S1 Table — (DOCX) [file pone.0228226.s001.docx]

**S1 Table.** Characteristics of breast tissue samples.

| **ID no.** | **Age range** | **Lobule** | | **Carcinoma** | | | | | **DCIS**  **AMG** |
| --- | --- | --- | --- | --- | --- | --- | --- | --- | --- |
|  |  | **Density** | **AMG** | **AMG** | **Grade** | **ER** | **PR** | **HER2** |  |
| BR01 | 60-64 | Low | + | + | III | + | + | - | 0 |
| BR02 | 65-69 | Medium | + | 0 | III | + | - | - | na |
| BR03 | 60-64 | None | na | 0 | II | + | + | - | 0 |
| BR04 | 60-64 | Medium | 0 | 0 | I | + | + | - | na |
| BR05 | 60-64 | Medium | 0 | 0 | I | + | + | - | na |
| BR06 | 55-59 | Low | 0 | + | III | + | + | - | na |
| BR07 | 55-59 | High | + | 0 | II | + | - | + | na |
| BR08 | 45-49 | High | ++ | na | III | + | - | - | na |
| BR09 | 50-54 | Low | + | 0 | II | - | + | - | na |
| BR10 | 50-54 | High | + | 0 | III | + | + | - | na |
| BR11 | 45-49 | High | + | na | III | + | - | - | na |
| BR12 | 35-39 | High | 0 | 0 | II | + | + | - | na |
| BR13 | 35-39 | High | ++ | 0 | III | + | + | - | na |
| BR14 | 65-69 | High | ++ | 0 | II | + | - | - | na |
| BR15 | 30-34 | Low | 0 | + | III | + | + | - | na |
| BR16 | 40-44 | High | + | 0 | III | + | + | - | na |
| BR17 | 40-44 | Medium | 0 | na | II | - | + | - | na |
| BR18 | 50-54 | High | + | + | III | + | + | - | 0 |
| BR19 | 55-59 | High | ++ | 0 | III | + | + | - | na |
| BR20 | 65-69 | Medium | ++ | 0 | III | + | - | - | na |
| BR21 | 50-54 | High | 0 | + | III | + | + | - | na |
| BR22 | 40-44 | High | + | 0 | III | + | + | - | na |
| BR23 | 60-64 | Low | + | ++ | I | + | + | - | na |
| BR24 | 60-64 | Low | 0 | + | II | - | + | + | na |
| BR25 | 55-59 | Medium | 0 | ++ | I | + | + | - | ++ |
| BR26 | 60-64 | Medium | 0 | 0 | III | + | + | - | na |
| BR27 | 60-64 | Medium | + | 0 | III | - | - | + | na |
| BR28 | 50-54 | Low | 0 | 0 | III | + | + | - | na |
| BR29 | 65-69 | Low | + | + | II | + | + | - | na |
| BR30 | 65-69 | High | ++ | + | II | + | + | - | na |
| BR31 | 60-64 | None | na | 0 | III | - | + | - | na |
| BR32 | 55-59 | Medium | + | 0 | III | + | - | + | na |
| BR33 | 55-59 | Low | 0 | 0 | II | + | + | - | na |
| BR34 | 45-49 | High | 0 | na | III | + | - | + | na |
| BR35 | 60-64 | Low | + | na | II | + | + | - | na |
| BR36 | 50-54 | High | 0 | 0 | II | + | + | - | na |
| BR37 | 40-44 | Medium | 0 | 0 | III | + | + | - | na |
| BR38 | 55-59 | Medium | 0 | 0 | II | + | - | - | na |
| BR39 | 50-54 | None | na | 0 | III | + | + | - | na |
| BR40 | 50-54 | High | 0 | 0 | II | - | + | - | na |
| BR41 | 50-54 | High | 0 | 0 | II | + | + | - | na |
| BR42 | 55-59 | Medium | ++ | 0 | III | + | - | + | na |
| BR43 | 60-64 | Low | + | 0 | III | - | + | - | na |
| BR44 | 65-69 | Medium | + | 0 | I | - | + | - | na |
| BR45 | 55-59 | Low | + | 0 | II | + | + | - | 0 |
| BR46 | 55-59 | Low | 0 | 0 | III | + | - | - | 0 |
| BR47 | 50-54 | High | 0 | 0 | II | + | + | - | 0 |
| BR48 | 55-59 | High | 0 | na | II | + | + | - | na |
| BR49 | 55-59 | High | + | 0 | II | + | - | - | na |
| BR50 | 50-54 | High | + | 0 | II | + | + | - | 0 |

AMG: autometallography, DCIS: ductal carcinoma in situ, ER: estrogen receptor, HER2: human epidermal growth factor receptor 2, na: not applicable, PR: progesterone receptor. AMG proportion: 0 none, + <5%, ++ ≥5%
