# Supplementary figures and images for: Elemental bioimaging shows mercury and other toxic metals in normal breast tissue and in breast cancers
Source: PLoS One. 2020 Jan 31;15(1):e0228226. doi: 10.1371/journal.pone.0228226 (PMC6993973; doi:10.1371/journal.pone.0228226)

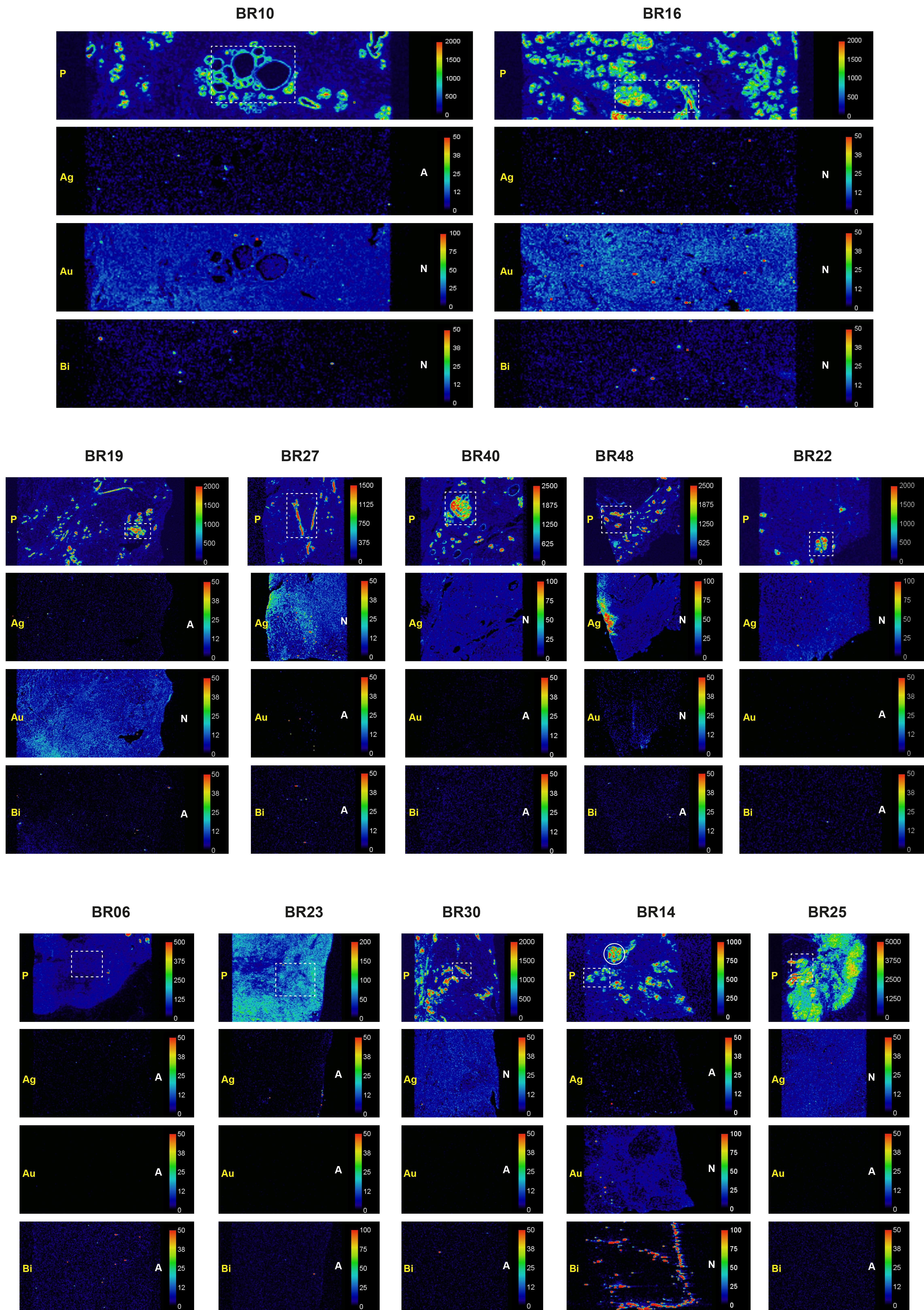

Supplement: S1 Fig — Breast samples showing the absence of localised staining for Ag, Au and Bi. Phosphorus (P) staining indicates cellular density. Selected regions stained with autometallography (AMG) are indicated in the dashed boxes. Element distribution = A: absent, N: non-localising. Scale = counts per second (proportional to abundance). (TIF) [file pone.0228226.s002.tif]
